# Immune modulation of Th1/Th2/Treg/Th17/Th9/Th21 cells in rabbits infected with *Eimeria stiedai*

**DOI:** 10.3389/fcimb.2023.1230689

**Published:** 2023-08-01

**Authors:** Xiao-Di Chen, Jing Xie, Yong Wei, Ji-Feng Yu, Ye Cao, Lu Xiao, Xue-Jing Wu, Cong-Jian Mao, Run-Min Kang, Yong-Gang Ye

**Affiliations:** ^1^ Key Laboratory of Animal Genetic and Breeding of Sichuan Province, Sichuan Animal Science Academy, Chengdu, China; ^2^ College of Animal and Veterinary Sciences, Southwest Minzu University, Chengdu, China

**Keywords:** *E. stiedai*, TBX2, GATA3, RORC, Foxp3, SPI1, BCL6

## Abstract

**Introduction:**

Despite long-term integrated control programs for Eimeria stiedai infection in China, hepatic coccidiosis in rabbits persists. Th1, Th2, Th17, Treg, Th9, and Th21 cells are involved in immune responses during pathogen infection. It is unclear whether Th cell subsets are also involved in E. stiedai infection. Their roles in the immunopathology of this infection remain unknown. Therefore, monitoring these T-cell subsets’ immune responses during primary infection of E. stiedai at both transcriptional (mRNA) and protein (cytokines) levels is essential.

**Methods:**

In experimentally infected New Zealand white rabbits, mRNA expression levels of their transcript—TBX2 (Th1), GATA3 (Th2), RORC (Th17), Foxp3 (Treg), SPI1 (Th9), and BCL6 (Th21)—were evaluated using quantitative real-time polymerase chain reaction (qRT-PCR), whereas Th1 (IFN-g and TNF-a), Th2 (IL4), Th17 (IL17A and IL6), Treg (IL10 and TGF-b1), Th9 (IL9), and Th21 (IL21) cytokines were measured using enzyme-linked immunosorbent assays (ELISAs).

**Results:**

We found that levels of TBX2, GATA3, RORC, SPI1, and BCL6 in the livers of infected rabbits were elevated on days 5 and 15 post-infection (PI). The concentrations of their distinctive cytokines IFN-g and TNF-a for Th1, IL4 for Th2, IL17A for Th17, IL9 for Th9, IL21 for Th21, and IL10 for Treg IL10 were also significantly increased on days 5 and 15 PI, respectively (p < 0.05). On day 23 PI, GATA3 with its cytokine IL4, RORC with IL17A, Foxp3 with IL10 and TGF-b1, and SPI1 with IL9 were significantly decreased, but TBX2 with IFN-g and IL6 remained elevated.

**Discussion:**

Our findings are the first evidence of Th1/Th2/Treg/Th17/Th9/Th21 changes in E. stiedai-infected rabbits and provide insights into immune regulation mechanisms and possible vaccine development.

## Introduction

Hepatic coccidiosis in rabbits is caused by *Eimeria stiedai*. First discovered more than 100 years ago, it has a high host specificity, infectivity, and lethality. Post-weaning rabbits are particularly susceptible, with mortality as high as 80% (Gomez-Bautista et al., 1987; Pakandl, 2009). Unlike the other 10 species of rabbit intestinal coccidia, *E. stiedai* lives in the rabbit liver to complete its entire development (Duszynski and Couch, 2013). The life cycle of *E. stiedai* occurs both in the external environment and within the host rabbit, and generally experiences three stages, *Sporogony*, *Schizogony* or *Merogony*, and *Gametogony*. Firstly, sporulated oocysts are consumed by rabbits and rupture, then released sporozoites invade the liver and bile duct epithelial cells where merogony occurs. Merozoites multiply within and are released from epithelial cells and continue to increase until they complete a repeat of at least four generations. Lastly, the life cycle enters the gametogony stage, macrogametes and microgametes combine into zygotes and eventually develop into oocysts passed out in the feces. *E. stiedai* causes severe damage to the hepatobiliary epithelial structure, affects the normal hepatobiliary function, leads to slow growth and high mortality in diseased rabbits (Oliveira et al., 2011; Jing et al., 2016), and causes severe harm to the rabbit industry. It is listed as a second-class animal disease by the Ministry of Agriculture of the People’s Republic of China.

T helper cells with specific cytokines (IFN-γ, IL4, IL10, IL17A, IL21, IL9, TNF-α, and TGF-β1) play essential roles in parasitic diseases (Pesce et al., 2006; Seki et al., 2012; Pang et al., 2014; Li et al., 2017; Kalantari et al., 2019; Bai et al., 2021; Al-Masoudi et al., 2021; Parhizgar et al., 2021; Li et al., 2022). Usually, interferon-γ (IFN-γ) is induced by *TBX2*, but in New Zealand white rabbits infected with *Schistosoma japonicum* (*S. japonicum*), it was found that the transcription factor *TBX2* was positively correlated to TNF-α, but not to IFN-γ, which was correlated to another Th1 transcriptional factor, *IRF8* (Farwa et al., 2018); thus, our study aimed to evaluate the correlation between T helper cells, transcription factors (*TBX2*, *GATA3*, *Foxp3*, *RORC*, *SPI1*, and *BCL6*), and cytokines (IFN-γ, IL4, IL10, IL17A, IL21, IL9, TNF-α, and TGF-β1) in rabbits infected with *E. stiedai*.

The Th1 cytokine IFN-γ sometimes protects against damage, while the Th2 cytokine IL4 enhances fibrogenesis (McManus et al., 2010; Du et al., 2011). Treg cells that produce IL10 cytokines play a crucial role in the immune balance; it acts as an immunosuppressant or a fibrotic inducer (Layland et al., 2010; Adalid-Peralta et al., 2011; Zhou et al., 2015). For example, in *Schistosoma mansoni*-infected mice, higher expression levels of hepatic IL17A were found in CBA mice, suggesting that its lesions may be associated with IL17A produced by Th17 cells (Kalantari et al., 2019). However, lacking the IL21/IL21R axis in *S. mansoni-*infected mice attenuated granulomatous inflammation and liver fibrosis formation in mice (Pesce et al., 2006). There were significantly increased levels of Th9 (IL9) in the liver of C57BL/6 mice infected by *S. japonica* in the early stage, suggesting that IL9 may be involved in liver injury (Li et al., 2017). However, whether these Th subsets and their cytokines also play an immuno-protective or damaging role in *E. stiedai* infection is unknown.

*TBX2* induces the production of interferon-γ (IFN-γ) and orchestrates the Th1 cell migration by regulating the expression of chemokines and chemokine receptors. *TBX2* and *GATA3* regulate Th1/Th2 cell fate using a synchronous mechanism, by directly binding to the genetic locus of cytokines: *TBX2* binds IFN-γ whereas *GATA3* binds IL4 and other Th2 cytokines such as IL5 and IL13, leading to the expression of these proteins (Kanhere et al., 2012).

Transcriptional factor *Foxp3* serves as a lineage specification factor of Treg cells, and is induced either during native Treg (nTreg) development in the thymus or in the presence of TGF-β1 and retinoic acid. High levels of IL17 is expressed by Th17 under the transcriptional regulation of *RORC.* In some studies, it was suggested that the signature transcription factor *RORC* of Th17 cells is also induced by TGF-β1, thus leading to differentiation of Treg and Th17 lineages. In the absence of a second signal derived from proinflammatory cytokines, *Foxp3* can inhibit *RORC* function and promote Treg differentiation. However, when cells also receive signals from proinflammatory cytokines (e.g., IL6), the function of *Foxp3* is inhibited and Th17 differentiation is induced. Thus, it is the balance between *Foxp3* and *RORC* function that determines the fate of CD4+T cells and the type of immune response that would be generated (Ziegler and Buckner, 2009; Ren and Li, 2017).

*PU.1* (*SPI1*) is a transcription factor that belongs to the E-twenty six (ETS) family of proteins and is a key factor for the differentiation of the IL9-producing Th9 subset, in humans and mice (Chang et al., 2010; Ramming et al., 2012). *BCL6* served as a master regulator of Th21 differentiation, and Th21 differentiation does not occur *in vivo* in the absence of *BCL6* (*BCL6* −*/*−) (Johnston et al., 2009; Nurieva et al., 2009; Yu et al., 2009). While *BCL6* is vital in both GC B cells and Th21 cells, it can control Th21 differentiation mainly by regulating genes of GC B cells (Crotty et al., 2010).

In *E. stiedai* infection, it is unclear whether different mechanisms are involved. For these reasons, we measured dynamic changes in Th cells, including Th1, Th2, Th17, Treg, Th9, and Th21, during different stages of *E. stiedai* infection. Our findings provide a theoretical basis to control for protective and therapeutic vaccinations.

## Materials and methods

### Ethics statement

This study was carried out in full compliance with the framework of noncommercial scientific research, authorized by the Animal Ethics Committee of Sichuan Animal Science Academy (approval no. AECSASA2023-18).

### Parasites

The *E*. *stiedai* strain in this study was provided by the Department of Parasitology at Sichuan Agricultural University. Three coccidia-free New Zealand white rabbits (5 weeks old) were orally infected with *E. stiedai* to obtain more oocysts. Then high numbers of unsporulated oocysts were harvested from liver with grinding, filtration, and centrifugation. Finally, the oocysts were sporulated in 2.5% potassium dichromate solution at 28°C and stored at 4°C.

### Experimental rabbits

We divided 36 female New Zealand white rabbits aged 50 days into a control group (*n* = 18, six rabbits for each time point) and an experiment group (*n* = 18, six rabbits for each time point). They received 1.0 × 10^5^ *E. stiedai* orally and were euthanized on days 5, 15, and 23 post-infection (PI). All rabbits were monitored for 5 days by testing the daily collected fecal samples to confirm the infection. Blood samples were collected for quantitative real-time polymerase chain reaction (qRT-PCR) and enzyme-linked immunosorbent assays (ELISAs).

### Blood sample collection

We collected 3 ml of blood from the rabbits on days 5, 15, and 23 PI. Serum was also collected and stored at −80°C to evaluate Th1/Th2/Treg/Th17/Th9/Th21-related cellular cytokines.

### RNA extraction and cDNA preparation

RNA was extracted from the isolated blood using the TRIzol method (TRIzol reagent provided by Invitrogen, Carlsbad, CA, USA), following the manufacturer’s protocols. cDNA preparation was performed in a 20-μl mixture condition using Hifar^®^ II 1st Strand cDNA Synthesis SuperMix for qRT-PCR (Yeasen Biotechnology Co., Ltd) as follows: 4 μl of RNA, 2 μl of 5× gDNA digester Buffer, 1 μl of gDNA, 10 μl of 2× Hifar^®^ II SuperMix plus, and 3 μl of sterile RNase-free water. The amplification conditions were 25°C for 5 min, 42°C for 30 min, and 85°C for 5 min.

### Primer design and qRT-PCR amplification

CDS sequences of *TBX2*, *GATA3*, *Foxp3*, *RORC*, *BCL6*, and *SPI1* genes and housekeeping *GAPDH* as a reference gene were retrieved from the GenBank database with their accession numbers as illustrated in C 1. For qRT-PCR expression profiles, primer pairs of each target gene were designed by Premier 6.0 software, where their length ranged from 18 to 25 bp with 55%–65% of GC content and different annealing temperatures (**Table 1**). All primers were synthesized by Tsingke Biotechnology (Chengdu) Co., Ltd. qRT-PCR was performed in a 20-μl mixture condition using Hieff UNICON^®^ Universal Blue qPCR SYBR Green Master Mix (Yeasen Biotechnology Co., Ltd) as follows: 1 μl of cDNA, 10 μl of Master Mix, 0.4 μl of forward primer, 0.4 μl of reverse primer, and 8.2 μl of sterile RNase-free water. The amplification conditions started with initial denaturation at 95°C for 2 min, followed by 40 cycles of denaturation at 95°C for 10 s, 57°C for 30 s at annealing temperature, and the melting curve stage at 95°C for 15 s, 55°C for 15 s, followed by 95°C for 15 s.

**Table 1:** Characteristics of primers used for qRT-PCR.

Primer name	Accession no.	Sequence (5′ → 3′)	*T*_m_ (°C)	Product size (bp)
**TBX2**	XM_008271188.1	Forward: CCAACGACATCCTCAAGCTG; reverse: TTGTCGTTCTGGTAGGCAGT	58	94
**GATA3**	XM_002717361.2	Forward: AGAAGGAAGGCATCCAGACC; reverse: AGGACATGTGTCTGGACAGG	58	135
**Foxp3**	XM_002719910.2	Forward: TTCCGAAACCACCCTGCTAC; reverse: AACTCATCCACGGTCCACAC	59	113
**RORC**	XM_008264444.2	Forward: ACCAGATCGTGCTCCTCAAA; reverse: ATGCCACCGTATTTGCCTTC	59	112
**BCL6**	XM_008266642.2	Forward: ATCAAAGCCAGTGAAGCGGA; reverse: AAAGGCTCTGCTCTCACACC	59	168
**SPI1**	XM_017348391.1	Forward: GTTCCCTACGACACGGATCT; reverse: TGTGGCTCTCTCCATCACTG	58	79
**GAPDH**	NM_001082253.1	Forward: TGACGACATCAAGAAGGTGGTG; reverse: GAAGGTGGAGGAGTGGGTGTC	60	121

### ELISA

According to the manufacturer’s instructions (Shanghai MLBIO Biotechnology Co. Ltd), an ELISA test was performed to measure nine cytokine levels—IFN-γ, TNF-α, IL4, IL10, TGF-β1 IL17A, IL6, IL9, and IL21—for the control and experiment group. Concentrations of plasma cytokines were calculated by using ELISA Calc v 0.1 software.

### Hepatic histopathology

Rabbits were euthanized on days 5, 15, and 23 PI. Partial left lobes of the livers from all rabbits were fixed in 10% neutral buffered formalin and embedded in paraffin for histological examination.

### Total hepatic oocysts

The total liver weight was determined by electronic weighing, then 10 g of the left liver lobe was ground, dissolved in 50 ml of physiological saline, and filtered with an 80-mesh filter screen. Counts were performed with a hemocytometer.

### Statistical analysis

The general linear model procedure in SPSS 22.0 version was used to analyze mRNA expression levels of *TBX2*, *GATA3*, *Foxp3*, *RORC*, *BCL6*, and *SPI1*, as well as plasma cytokine levels of IFN-γ, TNF-α, IL4, IL6, IL10, IL9, IL17, IL21, and TGF-β1. Differences were considered significantly different when *p* < 0.05. Regression and correlation analyses were performed to detect the relationships between each mRNA transcriptional factor and every cytokine individually. Another set of regression and correlation was performed to determine the relationships between different mRNA transcriptional factors and the relationships between transcriptional factors and oocyst data, all at three time points (on days 5, 15, and 23 PI). |*r*| values ≥ 0.8 were considered statistically significant.

## Results

### Hepatic histopathological changes

**Figure 1:**
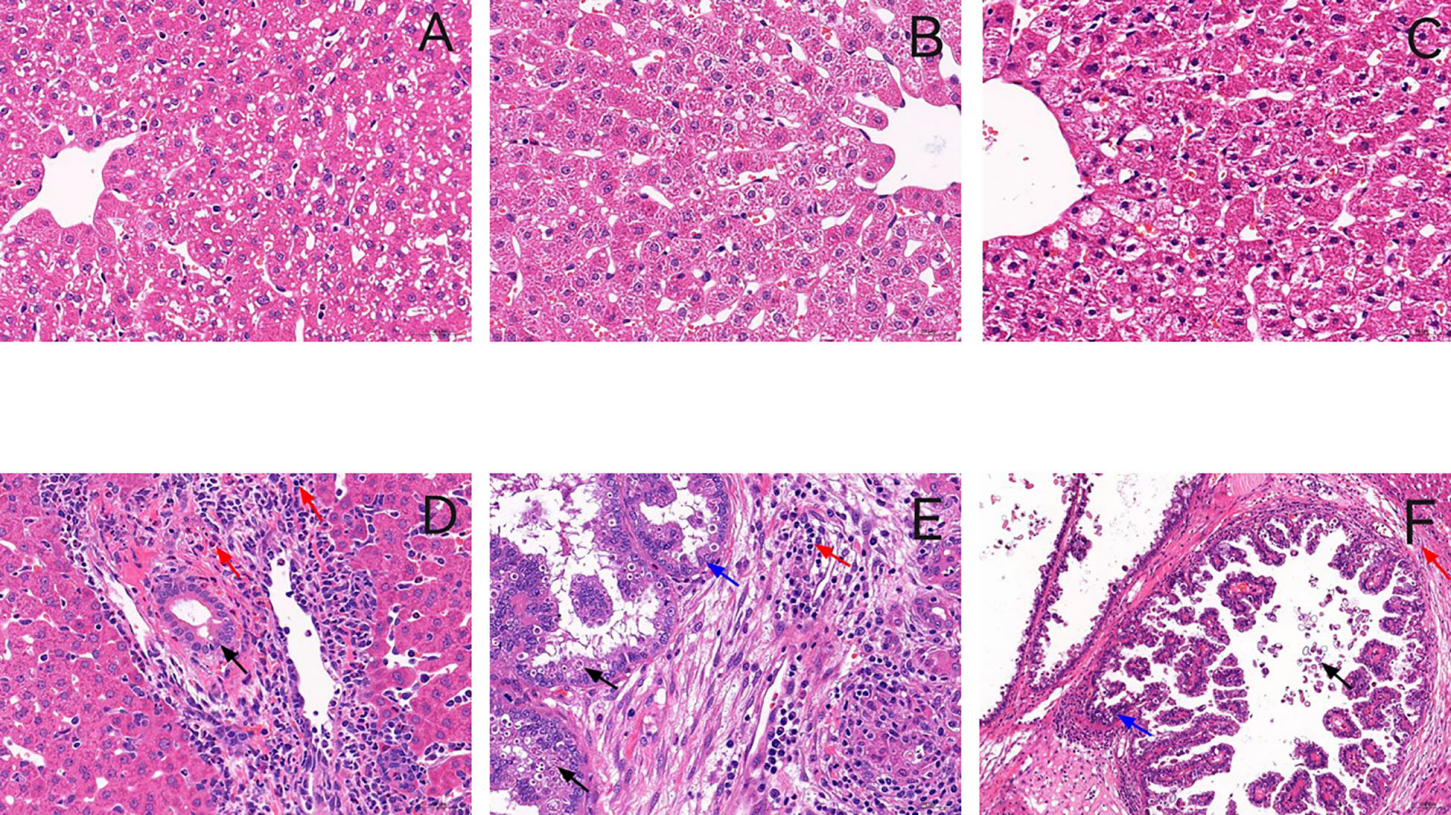
Representative histology of liver sections stained with hematoxylin and eosin. **(A–C)** show liver sections from the control group in three time points, and **(D–F)** show liver sections from the experiment group in three time points (on days 5, 15, and 23 PI) (×400). In the experiment group, massive inflammatory cells were infiltrated around the portal areas accompanied by cholangiocyte proliferation on day 5 PI **(D)**. The number of inflammatory cells increased, and bile duct hyperplasia was exaggerated on day 15 PI **(E)**. Many schizonts were observed (black arrow). On day 23 PI (post-infection) bile duct expansion, a proliferation of bile duct epithelial cells and fibrous tissue hyperplasia, and numerous *Eimeria stiedai* eggs appeared **(F)**.

No rabbit died during the infection period, and the rabbits were euthanized on days 5, 15, and 23 PI. The effect of *E. stiedai* infection on rabbit livers was investigated using stained paraffin-embedded tissues. In control group, as shown in **Figures 1A–C** and in the experiment group, as shown in **Figures 1D–F**, in the experiment group, substantial numbers of inflammatory cells had infiltrated around the portal area, collagen depositions were slightly stained, and intrahepatic bile duct epithelia were proliferated in the liver tissue. The intrahepatic bile ducts continued to be exaggerated extensively on days 15 and 23 PI (**Figures 1E, F**). Several merozoites were observed (black arrow) (**Figure 1E**). On day 23 PI, the proliferation of bile duct epithelial and numerous eggs of *E. stiedai* cells was observed (**Figure 1F**).

### T helper-1 response: *TBX2* mRNA expressions and IFN-γ and TNF-α serum levels

**Figure 2:**
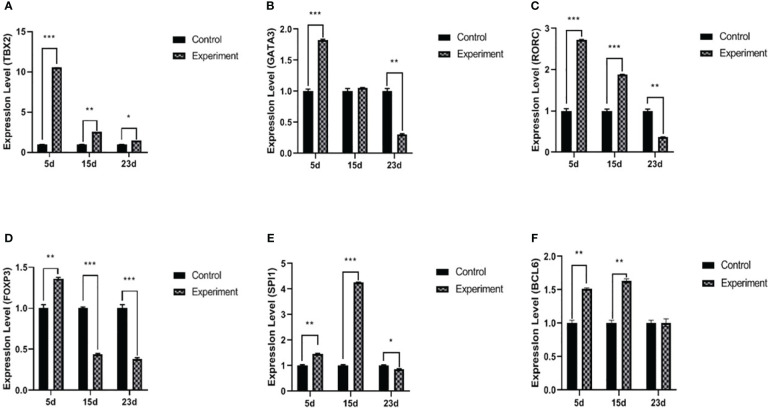
mRNA expression levels of *TBX2* **(A)**, *GATA3* **(B)**, *RORC* **(C)**, *Foxp3* **(D)**, *SPI1* **(E)**, and *BCL6* **(F)** transcriptional factors. The experiment group on days 5, 15, and 23 PI, compared with the control group, *p < 0.05, **p < 0.01, ***p < 0.001.

**Figure 3:**
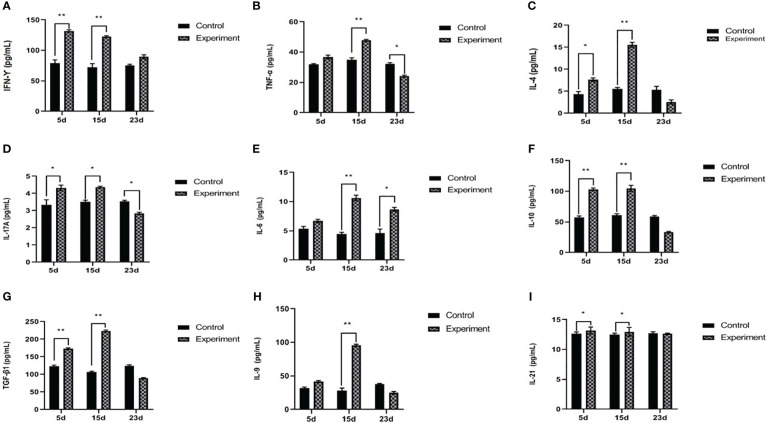
Serum concentration of IFN-γ **(A)**, TNF-α **(B)**, IL4 **(C)**, IL17A **(D)**, IL6 **(E)**, IL10 **(F)**, TGF-β1 **(G)**, IL9 **(H)**, and IL21 **(I)**. The experiment group on days 5, 15, and 23 PI, compared with the control group, *p < 0.05, **p < 0.01.

On days 5 and 15 PI, *TBX2* mRNA expressions and the levels of IFN-γ and TNF-α in serum were significantly increased after the infection compared to the control group. However, on day 23 PI, the infected rabbits showed a significant decrease in TNF-α (*p* < 0.05), whereas *TBX2* mRNA (*p* < 0.05) expression and IFN-γ (*p* > 0.05) were elevated on day 23 PI (**Figures 2A**, **3A, B**).

### T helper-2 response: *GATA3* expressions and IL4 serum levels

On day 5 PI, *GATA3* mRNA and IL4 significantly increased after the infection compared to the control. Furthermore, on day 15 PI, IL4 was significantly increased (*p* < 0.01) without any effect on *GATA3* mRNA. On day 23 PI, the infected group showed a decrease in *GATA3* (*p* < 0.01), and IL4 was not significantly different (*p* > 0.05) (**Figures 2B**, **3C**).

### T helper-17 response: *RORC* mRNA expressions and IL17A and IL6 serum levels

The levels of *RORC* mRNA (*p* < 0.001) and IL17A (*p* < 0.01) in serum were significantly increased on day 5 PI, but there was no statistical difference in the IL6 serum levels. On day 15 PI, *RORC* mRNA (*p* < 0.001), IL17A (*p* < 0.05), and IL6 (*p* < 0.01) were significantly upregulated. On day 23 PI, RORC mRNA (*p* < 0.01) and IL17A (*p* < 0.05) oppositely decreased. Interestingly, IL6 serum levels were upregulated during the three periods in the experiment groups (**Figures 2C**, **3D, E**).

### T regulatory response: *Foxp3* mRNA expressions and IL10 and TGF-β1 serum levels

The level of *Foxp3* was significantly increased (*p* < 0.001) on day 5 PI, and IL10 and TGF-β1 were also significantly increased (*p* < 0.01), compared with normal control. Interestingly, on day 15 PI, the level of *Foxp3* was significantly decreased (*p* < 0.001); however, IL10 and TGF-β1 were still significantly increased (*p* < 0.01) compared with normal control. On day 23 PI, the levels of *Foxp3* and relative cytokines (IL10 and TGF-β1) in serum were significantly decreased (*p* < 0.05) (**Figures 2D**, **3F, G**).

### T helper-9 response: *SPI1* mRNA expressions and IL9 serum levels

On day 5 PI, the level of *SPI1* mRNA was significantly increased (*p* < 0.01); however, there was no statistical difference in IL9 (*p* > 0.05). On day 15 PI, *SPI1* mRNA (*p* < 0.001) and IL9 (*p* < 0.01) were significantly elevated. On day 23 PI, the level of *SPI1* mRNA was significantly decreased (*p* < 0.05), but there was no statistical difference in the concentration of IL9 between the normal control and infected groups (**Figures 2E**, **3H**).

### T helper-21 response: *BCL6* mRNA expressions and IL21 serum levels

*BCL6* mRNA and IL21 were increased with the infection (*p* < 0.01 and *p* < 0.05, respectively) on day 5 PI. This influence was relatively stable on day 15 PI (*BCL6*, *p* < 0.01 and IL21, *p* < 0.05). On day 23 PI, the levels of *BCL6* mRNA and IL21 showed no statistical difference between the normal control and infected group (*p* > 0.05) (**Figures 2F**, **3I**).

### Relationships between different transcriptional factors and their cytokines

There are relationships between Th1/Th2/Th17/Treg/Th9/Th21 transcriptional factors with their cytokine lineages, where positive correlations (*r* ≥ 0.8) were found between *RORC* and IL17A, *SPI1* and IL9, and *BCL6* and IL21 in the experiment groups throughout the whole period of infection as shown in **Table 2**. *TBX2* was positively correlated to TNF-α on days 5 and 15 PI. Cross-correlations were detected between transcriptional factors and cytokines of other T helper subsets, where *TBX2* was negatively correlated to IL10 on day 5 PI. *GATA3* was positively correlated to IL6 during the whole period of infection. *GATA3* was negatively correlated to IL4 on day 5 PI. *Foxp3* reported a positive correlation with IL4 and TGF-β1 (*r* ≥ 0.8) on day 5 PI, and *RORC* negatively correlated to TGF-β1 on days 15 and 23 PI.

**Table 2:** Correlation between transcriptional factors and their cytokines.

		TBX2	GATA3	RORC	Foxp3	SPI1	BCL6
5 days	IFN-γ	—	—	—	—	—	—
	TNF-α	0.998	—	—	—	—	—
	IL4	—	—	—	0.817	—	—
	IL17A	—	—	0.904	—	—	—
	IL6	—	0.807	—	—	—	—
	IL10	-0.900	—	—	—	—	—
	TGF-β1	—	—	—	0.964	—	—
	IL9	—	—	—	—	0.897	—
	IL21	—	—	—	—	—	0.999
15 days	IFN-γ		—	—	—	—	—
	TNF-α	0.800	—	—	—	—	—
	IL4	—	-0.976	—	—	—	—
	IL17A	—	—	0.989	—	—	—
	IL6	—	0.821	—	—	—	—
	IL10	—	—	—	—	—	—
	TGF-β1	—	—	-0.954	—	—	—
	IL9	—	—	—	—	0.858	—
	IL21	—	—	—	—	—	0.897
23 days	IFN-γ	—	—	—	—	—	—
	TNF-α	0.8113	—	—	—	—	—
	IL4	—	—	—	—	—	—
	IL17A	—	—	0.863	—	—	—
	IL6	—	0.884	—	—	—	—
	IL10	—	—	—	—	—	—
	TGF-β1	—	—	-0.996	—	—	—
	IL9	—	—	—	—	0.811	—
	IL21	—	—	—	—	—	0.812

**Figure 4:**
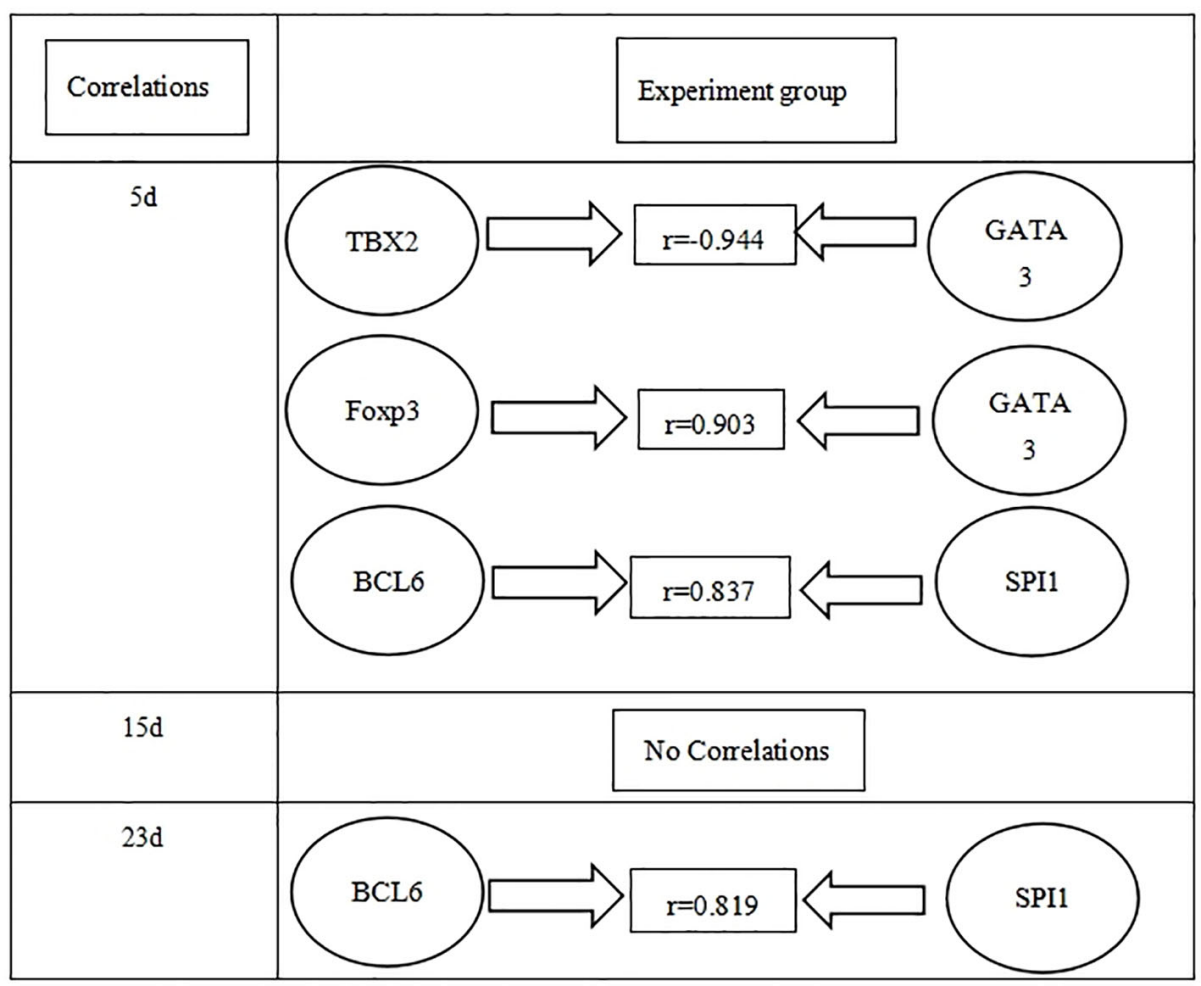
Correlations between different transcriptional factors in the experiment group in three time points (days 5, 15, and 23 PI).

The relationships between different transcriptional factors are illustrated in **Figure 4**.

Hepatic weight and oocyst data are illustrated in **Table 3**.

**Table 3:** Liver weight and oocysts data in the experiment group at three time points.

Number/Infection time/Liver weight (g/Oocyst data		1#	2#	3#	4#	5#	6#
**5 days**	Liver weight(g)	50.21	49.54	48.34	47.51	49.19	47.55
	Oocyst data	0	0	0	0	0	0
**15 days**	Liver weight(g)	81.85	84.89	84.79	74.72	70.39	86.71
	Oocyst data	1.49×10^6^	1.51×10^6^	1.50×10^6^	1.34×10^6^	1.27×10^6^	1.53×10^6^
**23 days**	Liver weight(g)	274.24	242.27	235.96	233.75	200.00	257.00
	Oocyst data	1.43×10^9^	1.39×10^9^	1.27×10^9^	1.26×10^9^	1.02×10^9^	1.40×10^9^

Correlation between transcriptional factors and oocyst data are illustrated in **Table 4**.

**Table 4:** Correlation between transcriptional factors and oocysts data.

		TBX2	GATA3	RORC	Foxp3	SPI1	BCL6
**Oocysts**	**5 days**	—	—	—	—	—	—
	**15 days**	—	—	—	—	—	—
	**23 days**	—	—	−0.968	−0.908	−0.935	—

## Discussion

We studied dynamic changes in Th mRNA expression levels of their transcript—*TBX2* (Th1), *GATA3* (Th2), *RORC* (Th17), *Foxp3* (Treg), *SPI1* (Th9), and *BCL6* (Th21)—and analyzed the potential effects of their imbalance on disease outcomes in the rabbit. Moreover, *E. stiedai* infections provide an excellent model to study the roles of Th cell transcriptional factors. In the early stage of the infection with *E. stiedai* (on day 5 PI), Th1 dominated at both transcriptional and protein levels. We also found a Treg response in the late stage of infection. Furthermore, our study also showed that Th17, Th9, and Th21 were involved in *E. stiedai* infection. However, helminth infection is characterized by Th2-dominated immune responses, which may contribute to chronic infection and the host’s inability to clear the parasites (Kim et al., 2012; Uddin et al., 2012). For Th1, the transcription factor *TBX2* was positively correlated to TNF-α, but not IFN-γ, which Farwa found was correlated to another Th1 transcriptional factor IRF8 (Farwa et al., 2018). Mechanistically, *TBX2-*dependent innate IFN-γ is essential in *T. gondii* infection T-bet to produce IFN-γ, which is essential for host resistance to intracellular infection by maintaining IRF8 inflammatory dendritic cells at the infection site (Lopez-Yglesias et al., 2021). A report showed that loss of *TBX2* had some effect on IFN-γ production in T cells but not parasite-specific T cells (Harms Pritchard et al., 2015).

For Th2, the expression levels of *GATA3* and IL4 appeared to be elevated on days 5 and 15 PI, and *GATA3* mRNA expression was uncorrelated to IL4, similar to *GATA3* mRNA expression, which was essential for IL13 production but not IL4 in the experiment group, whereas IL4 appeared to be a STAT6-dependent stimulation (Zhu, 2010). Regarding Treg, the expression levels of *Foxp3* also appeared to be elevated. TGF-β1 was also induced simultaneously, similar to an observation by Tang et al. (2015) on day 5 PI. *Foxp3* appeared to have elevated expression levels, and the same result appeared in *S. japonicum* infection, which was a potent inducer for *Foxp3* (Finlay et al., 2014). For Th17, *RORC* expression was correlated to IL17A. On days 5 and 15 PI, Th17 mRNA and IL17A expression levels were elevated, similarly in *S. mansoni*-infected mice, in which the liver developed significant hepatic granulomatous inflammation, with higher expression levels of IL17 and *RORC*, suggesting that its lesions may be associated with IL17 produced by Th 17 cells (Kalantari et al., 2019). For Th9, the expression levels of *SPI1* and IL9 appeared to be elevated on days 5 and 15 PI, and *SPI1* expression was correlated to IL9. The expression of IL9 and its transcription factor *SPI1* in hydatid patients was significantly higher in focal tissue, suggesting the formation of chronic granuloma in hydatid by upregulating the expression of Th9 cells, IL9, and transcription factor *SPI1* during infection (Tuxun et al., 2015). The expression levels of Th21, *BCL6*, and IL21 appeared to be elevated on days 5 and 15 PI, and *BCL6* expression was correlated to IL21. A correlation study of IL21 and transcription factor *BCL6* showed that IL21 was positively correlated to *BCL6*, which was upregulated on Th21 cells, and IL21 is the primary effector molecule of Tfh cells. (Geng et al., 2018).

In addition, many cross-modulations were observed; TBX2 suppressed *GATA3* expression on day 5 PI (Zhu, 2010). Cross-modulations between *GATA3* and *Foxp3* were observed in Treg cells, where *GATA3* expression enhanced the accumulation of Treg in the inflammatory site (Turner et al., 2011). On days 5 and 23 PI, we also observed the cross-modulations between *SPI1* and *BCL6.* The expression of B-cell lymphoma protein 6 (*BCL6*) depends on *IRF8* and *SPI1 in vivo*, which is essential for developing germinal center B cells, and *SPI1* acts as a regulator of *BCL6* expression during GC B-cell development (Wang et al., 2019). *GATA3* promotes Th2 differentiation while inhibiting Th1, and *TBX2* has been shown to promote the development of Th1 and IFN-γ production (Szabo et al., 2000), but a study contradicted this finding (Cobb et al., 2010). A study showed that the IL21/IL21R signaling axis regulated the effector functions of T-cell subsets (e.g., production of cytokines) by enhancing *TBX2* and *STAT4* expression in human T cells, thereby enhancing IFN-γ production (Solaymani-Mohammadi et al., 2019). Treg only regulated TGF-β1 and was independent of type 2 cytokines (Mishra et al., 2014). In general, IL6 combined with TGF-β induced differentiation of Th17 cells from initial CD4+T cells and was also polarized into Treg cells after co-stimulation with IL2 and TGF-β (Kimura and Kishimoto, 2010). Our study found interesting findings: *GATA3*, *Foxp3*, *RORC*, and *SPI1* mRNA and its relative cytokines (such as IL4, IL10, IL17A, and IL9 in the serum) were significantly decreased in the late stage, which suggests that they were suppressed.

In the experiment group, liver weight increased, and the significant proliferation of eggs of *E. stiedai* made liver lesions severe (**Table 3**). In the liver sections, massive inflammatory cells were infiltrated around the portal areas accompanied by cholangiocyte proliferation on day 5 PI (**Figure 1D**). *TBX2*, *GATA3*, *Foxp3*, *RORC, BCL6, SPI1*, and relative cytokine expression levels were elevated, which resisted *E. stiedai* infection. Several merozoites were observed on day 15 PI (**Figure 1E**) (black arrow). *TBX2*, *RORC, BCL6, SPI1*, and relative cytokine expression levels were elevated, and *Foxp3* expression levels were decreased, likely due to dynamic Treg/Th17 changes. On day 23 PI, the appearance of numerous eggs of *E. stiedai* was consistent with previous reports (**Figure 1F**), except *TBX2* expression levels were elevated; *GATA3, RORC, BCL6*, and *SPI1* expression levels were decreased; and Th1 dominated at both transcriptional and protein levels, but this contradicted a study carried out by Uddin et al. (2012). In addition, *RORC*, *Foxp3*, and SPI1 were negatively correlated to oocyst numbers on day 23 PI, and expression levels of cytokines including IL17A, IL10, IL9, and TGF-β1 decreased. No statistically significant difference of relationships between *TBX2*, *GATA3, BCL6*, and oocyst output was observed in the experiment groups at three time points (**Table 4**). In the experiment group, the severe liver damage was observed during the proliferation of *E. stiedai*, likely through suppressing the expression of *RORC*, *Foxp3*, *SPI1*, cytokines (IL17A, IL10, and IL9), and TGF-β1.

## Conclusion

Our findings provide the first evidence of dynamic changes in Th1/Th2/Th17/Treg/Th9/Th21 and confirm an imbalance of these cell populations in *E. stiedai-*infected rabbits. Additional studies are needed to elucidate the detailed roles of these Th cells in the pathogenesis of *E. stiedai*.

## Data availability statement

The raw data supporting the conclusions of this article will be made available by the authors, without undue reservation.

## Ethics statement

The animal study was reviewed and approved by the Institutional Animal Care Committee of Sichuan Animal Science Academy.

## Author contributions

Conceptualization: X-DC, R-MK, and Y-GY. Methodology: X-DC. Software: LX, C-JM, and X-JW. Validation: J-FY, YW, and JX. Formal analysis: X-DC and YC. Resources: Y-GY. Data curation: R-MK and Y-GY. Writing—original draft preparation: X-DC. Writing—review and editing: Y-GY. Visualization: X-DC. Supervision: Y-GY. Project administration: R-MK and Y-GY. All authors contributed to the article and approved the submitted version.
